# Socio-ecological predictors of HIV testing in women of childbearing age in Nigeria

**DOI:** 10.11604/pamj.2022.41.162.30345

**Published:** 2022-02-23

**Authors:** Hassana Bashir Yakasai, Bashir Adam Yakasai

**Affiliations:** 1 461 Nigerian Airforce Hospital, Kaduna, Nigeria,; 2 Kaduna State University, Kaduna, Nigeria

**Keywords:** HIV testing, childbearing, women, HIV prevention, Nigeria

## Abstract

**Introduction:**

HIV remains a public health problem in Nigeria. Women within the age of 15 to 49 years, the childbearing age, have a prevalence rate of 1.9%, higher than that of their male counterparts of the same age group. Women can transmit HIV to their partners and their children. Nigeria accounts for 30% of global transmission of HIV from mother to child. Therefore, the study seeks to identify the socio-ecological predictors of HIV testing because HIV testing is the gateway to HIV prevention to achieve the sustainable development goal of zero new infections by the year 2030.

**Methods:**

the study was a cross-sectional study, analyzing the 2013 Nigeria demographic and health survey data using SPSS V27.

**Results:**

the result of the study indicated a higher odds ratio for good comprehensive knowledge of HIV (p<0.001, OR=3.81), good attitude to HIV (p<0.001, OR=2.50), and high perceived risk of HIV (p<0.001, OR=2.03). A low odds ratio was observed for good cultural belief despite the significance of the association (p<0.001, OR=0.83).

**Conclusion:**

socio-ecological factors of HIV knowledge, attitude to HIV, perceived risk, and cultural belief were significant predictors of HIV testing in women of childbearing age. Programs targeted at women aged 15-19 years will enhance HIV testing as the gateway to HIV prevention and achieve the 95-95-95 target and zero new infections by 2030.

## Introduction

Human immunodeficiency virus (HIV) infection is still a public health problem associated with morbidity and mortality. Sub-Saharan Africa bears the highest burden of HIV, and Nigeria is said to have the second-highest burden of HIV, second to South Africa [1]. About 37.9 million people lived with HIV in 2018 globally [2]. It was estimated that 6,000 women aged 15-24 years become infected with HIV every week globally. Young women aged 1524 years in sub-Saharan Africa were twice more likely to be living with HIV than their male counterparts [2]. In Nigeria, the prevalence of HIV in women of childbearing age (defined as 15-49 years) is 1.9%. This is higher than that of males of the same age group (0.9%) [3]. Heterosexual transmission is the most prevalent route of HIV transmission in Nigeria. However, Nigeria accounts for 30% of mother-to-child transmission of HIV globally [1,4].

HIV testing is the entry point for HIV treatment and Prevention of Mother-to-Child Transmission (PMTCT). These are measures that can lead to the elimination of new HIV infections. Studies have shown low HIV testing in Nigeria. HIV testing rate of 30.4% among youths was found by [5], while a study by [6] revealed a testing rate of 15% among adolescents in Nigeria. An HIV testing rate of 36.3% was found among pregnant women in Western Nigeria [7], while an HIV testing rate of 20.7% was found among pregnant women in Northern Nigeria [8]. The result of the 2018 Nigeria HIV/AIDS Indicator and Impact Survey (NAIIS) revealed that out of the 1.9 million people living with HIV in Nigeria, only 67% were aware of their HIV status, about half of them (52%) were on antiretroviral therapy, and only 42% had viral suppression [3]. Nonetheless, Okeafor and Okeafor found that the rate of HIV testing among women aged 15-49 years who participated in the national demographic and health survey of 2003 and 2015 increased from 17.4% to 21.7% [9]. Notwithstanding, the increase is still low compared to the target of 95%.

Some socio-ecological factors studied in HIV testing are stigma, socioeconomic status, the perceived barrier to HIV services, perceived HIV risk, comprehensive knowledge of HIV, religious and sociocultural factors. Hoang *et al*. found that educational status, occupation, and source of HIV information were associated with knowledge and attitude towards HIV, including HIV testing in Vietnam [10]. Among people who inject drugs in Iran, Noroozi *et al*. found that HIV testing was associated with education, income, perceived risk of HIV infection, and lower level of HIV-related stigma [11]. Socioeconomic status was significantly associated with HIV testing in Paris and Nigeria [12-14]. Lépine *et al*. found that wealth, education, HIV knowledge, stigma, and perceived risk were predictors of HIV testing in women in a relationship with men in Nigeria [13]. In a review of Nigeria DHS 2013 data, Asaolu *et al*. observed a statistically significant association between higher education, higher wealth index, having comprehensive HIV knowledge, higher HIV risk perception in terms of having more than one sexual partner and having sexually transmitted disease in the past one year with HIV testing among youths in Nigeria [15]. Ayodele studied university students and found that perceived HIV risk was significantly associated with HIV testing [16]. Women with higher education, less stigma towards people living with HIV have adequate HIV knowledge, and recognized a higher risk for HIV were more likely to test for HIV [13]. Gunn *et al*. in a review of HIV testing by women attending antenatal care, using the 2013 National Demographic and Health (NDHS), found that stigma, wealth index, and educational status were significantly associated with HIV testing by pregnant women in the Ante-Natal Care (ANC) [12]. Women with higher education, higher wealth index and less stigma were more likely to test for HIV. Bibiana *et al*. in a study of women of the reproductive age group in a community in Nigeria found a significant association between occupation and level of education with knowledge of voluntary counseling and testing of HIV [17].

There is a need to strengthen HIV testing in women of childbearing age. Socio-ecological factors that affect HIV testing have been studied in youths of various groups and also pregnant women. Little literature exists about the socio-ecological factors that affect HIV testing in women of childbearing age at the national level in Nigeria. Therefore, we intend to fill this gap by studying socio-ecological predictors of HIV testing (knowledge of HIV/AIDS, attitudes about HIV/AIDS, perceived risk of HIV/AIDS, cultural beliefs about HIV/AIDS) among women aged 15-49 years nationwide in Nigeria. Understanding the socio-ecological factors that predict HIV testing among these women will provide a basis for planning intervention strategies to improve HIV testing in them with the long-term aim of eliminating new HIV infections. While Lepine *et al*. studied factors that affected HIV testing among couples using the 2008 National Demographic and Health Survey (NDHS) data [13], this present study used a more recent dataset (2013 NDHS) focused on women within the age 15-49 years. The socio-ecological model of health promotion provided the framework for this study. This study aims to determine to what extent is there an association between socio-ecological factors (knowledge of HIV/AIDS, attitudes about HIV/AIDS, perceived risk of HIV/AIDS, cultural beliefs about HIV/AIDS) and HIV testing among women of childbearing age in Nigeria.

## Methods

The study was a quantitative secondary data analysis utilizing the 2013 Nigeria Demographic and Health Survey (NDHS) dataset. The 2013 NDHS is a national cross-sectional survey that involved sampling of households. Nigeria is divided administratively into 36 states and 774 local government areas. The sample of the households was selected via a 3-stage stratified cluster design with samples taken from urban and rural areas of the 774 local government areas of Nigeria. The population for the survey was women aged 15-49 years and men aged 15-49 years resident in the selected households. Thirty-eight thousand nine hundred forty-eight (38,948) women aged 15-49 years were interviewed between February to June 2013 using the women questionnaire in the 2013 NDHS. This study´s target population is female respondents aged 15-49 years who participated in the 2013 NDHS. The association between the socio-ecological factors and HIV testing among these women was examined and analyzed., The minimum sample size calculated using the G Power (3.1.9.4) calculator for this research was 652. However, all the women with complete information in the dataset were included for analysis.

The independent (outcome) variables are knowledge of HIV/AIDS, attitudes about HIV/AIDS, perceived risk of HIV/AIDS, and cultural belief, while the dependent (predictor) variable is HIV testing. The research aimed to determine how the independent variables predict HIV testing in women of childbearing age (15-49 years). HIV testing: The dependent variable is HIV testing. The variable “Ever tested for HIV” in the 2013 NDHS dataset was used as HIV testing variable. The “Ever tested for HIV” variable has a dichotomous response of “yes” or “no” and it was used as such.

**Knowledge of HIV/AIDS:** in the women’s questionnaire used for 2013 NDHS, there were questions about HIV/AIDS knowledge. The responses of the seven questions were scored to get the HIV related knowledge score used for analysis. The questions “Ever heard of AIDS, know a place to get an HIV test, reduce the risk of getting HIV: always use condoms, to reduce the risk of getting HIV: have one sex partner only, who has no other partners, a healthy-looking person can have HIV” have a “yes”, “no” or “don´t know” options. The “don´t know” option was recoded as “no”. The “Yes” option was coded as “1” and “No” was coded as “0”. For the questions: “Can get HIV from mosquito-bite and Can get HIV by sharing food with a person who has AIDS”, “No” was coded as “1” and “Yes” was coded as “0” because the questions tested the ability to reject misconceptions about HIV. The total score for knowledge of HIV ranged from 0-7, and it was further categorized into low knowledge (score of 0-6) and the score of seven as high knowledge.

**Attitudes about HIV/AIDS:** there were questions in the women´s questionnaire that reflect the attitude of the respondents to HIV. The respondents that agree to the question “Willing to care for a relative with AIDS, a female teacher infected with HIV, but is not sick, should be allowed to continue teaching, and would buy vegetable from a vendor” were coded as “yes” and scored “1”, those that answered “no” were scored “0”. Respondents that disagreed with the questions “People with HIV should be ashamed of themselves, and people with HIV should be blamed for bringing disease to the community” were coded as “yes” and scored “1” while that that agreed were scored “0”. The total score for attitude about HIV ranged from 0-5, and it was further categorized into negative attitude (score of 0-2) and positive attitude (score of 3-5). Perceived risk of HIV/AIDS: there were questions relating to HIV risk in the women´s questionnaire used for the 2013 NDHS. A positive response to the questions “Had any STI in the last 12 months, had genital sore/ulcer in last 12 months, had genital discharge in the last 12 months, had more than one sex partner including a spouse in the last 12 months” and negative response to the questions “Condom used during last sex with a most recent partner and Used condom every time had sex with a most recent partner in the last 12 months” were coded as “1”, the opposite response was coded as “0”. The total score perceived risk HIV ranged from 0-5, and it was further categorized into low risk (score of 0-1), medium risk (2) and high risk (score of 3-5).

**Cultural belief about HIV:** the response to the variable “Can get HIV by witchcraft or supernatural means,” which is dichotomous as “Yes” or “No,” was used as such as a cultural belief variable in the study. The “yes” response was recoded as bad cultural belief and the “no” response as good cultural belief.

Data analysis was done by the use of SPSS software version 27. Descriptive statistics and Chi square was used to summarize the respondents´ characteristics and the significance of the association between the predictor and outcome variables. At the same time, binary logistic regression was employed to analyze the predictive effects of knowledge of HIV/AIDS, attitudes about HIV/AIDS, perceived risk of HIV/AIDS, and cultural beliefs with HIV testing. The study was approved by Walden University institutional review board. The IRB approval number is 07-17-20-0544515.

## Results

Out of the 38,948 women that participated in the survey, 0.01% of the respondents had missing data for the dependent variable, which is HIV testing. Twenty-one percent (21.2%) of the women had missing data for knowledge of HIV/AIDS. More than nineteen percent (19.8%) of the respondents had missing data for attitude to HIV/AIDS. Twenty-seven percent (27%) had missing data for the perceived risk of HIV/AIDS, 19.4% of the respondents had missing data for the cultural belief of HIV. The cases with complete data on all the independent and the dependent variables were selected for analysis using logistic regression. A final sample size of 17,467 women was used for analysis.

Table 1 shows the sociodemographic characteristics of the 17,467 women aged 15-49 years that participated in the 2013 Nigeria demographic and health survey. The result showed that the highest proportion of the women (38.5%) were between the ages of 25-34 years and less than 10% were above 45 years. The majority of the women (84.9%) were married. Above half of the women (59.4%) lived in rural areas, while 40.6% lived in urban areas. Most of the respondents either had no education (35%) or only had secondary education (33.7%). Only 11.8% higher than secondary education. 70% of the respondents were working at the time of the survey.

**Table 1:** sociodemographic characteristics of the respondents

Sociodemographic variables	Frequency (n)	Percentage (%)
**Age distribution (years)**		
15-24	4453	25.5
25-34	6728	38.5
35-44	4602	26.3
45-49	1684	9.6
**Marital status**		
Single	1676	9.6
Married	14825	84.8
Living with partner	481	2.8
Widowed	186	1.1
Divorced	137	0.8
Separated	162	0.9
**Place of residence**		
Urban	7086	39.9
Rural	10381	60.1
**Level of Education**		
No education	6107	35.0
Primary	3406	19.5
Secondary	5890	33.7
Higher	2064	11.8

N=17467

The findings also highlighted that 41.4% of the respondents had tested for HIV while 58.6% had never tested for HIV at the survey time. Most of the respondents (67 %) had low knowledge of HIV, but only about one-quarter of the respondents (24.1%) had a good attitude toward HIV. Most women (79%) also had a good cultural belief about HIV. The result also showed that most of the women (95.9%) had a low risk of HIV. Table 2 shows HIV testing uptake by socio-ecological variables. Binary logistic regression was performed by manually matching each independent variable with the dependent variable in different models. After which, all the independent variables were examined against the dependent variable in a model to determine the socio-ecological predictors of HIV testing.

**Table 2:** HIV testing uptake by socio-ecological variables

Outcome variable			HIV testing status				
	Total		Never tested		Ever tested		p-value
	n	%	n	%	n	%	
**Knowledge of HIV/AIDS**							
Good	5780	33.0	2058	20.1	3704	51.1	<0.001
Inadequate	11707	67.0	8171	79.9	3538	48.9	
**Attitude to HIV**							
Good	4209	24.1	1614	15.8	2595	34.9	<0.001
Bad	13258	75.9	8613	84.2	4845	65.1	
**Perceived risk of HIV**							
High	214	1.23	82	0.80	132	1.82	<0.001
Medium	507	2.90	217	2.12	290	4.00	
Low	16746	95.8	9928	97.08	6818	94.17	
**Cultural belief about HIV**							
Good	13802	79.0	8042	78.6	5780	79.6	0.140
Bad	3665	21.0	2185	21.4	1480	20.4	

HIV: Human Immunodeficiency Virus; AIDS: Acquired Immunodeficiency Syndrome

**Knowledge about HIV/AIDS:** results from the logistic regression test indicated that Knowledge about HIV/AIDS is significantly associated with HIV testing; X^2^ (1, N = 17467) = 185.148, p < 0.001. The result also showed that knowledge of HIV is a predictor of HIV testing (OR=4.16, 95% CI 3.89 - 4.45, P < 0.001), indicating the model was able to distinguish between participant HIV testing. The result also showed that knowledge of HIV explained 1.0% (Cox & Snell R2) to 13.5% (Nagelkerke R2) of the variance in HIV testing by women aged 15-49 years. Furthermore, the model correctly classified 68% of the cases by HIV testing status.

**Attitude about HIV:** results from the logistic regression test indicated a statistically significant association between attitude to HIV/AIDS and HIV testing; X^2^ (1, N = 17467) = 923.423, p < 0.001. Attitude to HIV/AIDS is also a predictor of HIV testing (OR=2.98, 95% CI 2.78 - 3.20, p < 0.001), indicating the model was able to distinguish between participant HIV testing. The result also showed that attitude about HIV explained 5.1% (Cox & Snell R2) to 6.9% (Nagelkerke R2) of the variance in HIV testing by women aged 15-49 years. Furthermore, the model correctly classified 64.2% of the cases by HIV testing status.

**Perceived risk of HIV:** results from the logistic regression test indicated a statistically significant association between perceived risk of HIV/AIDS and HIV testing; X^2^ (2, N = 17467) = 89.961, p < 0.001. The result also showed that the perceived risk of HIV explained 5% (Cox & Snell R2) to 7% (Nagelkerke R2) of the variance in HIV testing by women aged 15-49 years. Furthermore, the model correctly classified 59.3% of the cases by HIV testing. Medium and high perceived risks were predictive of HIV testing (OR=1.95, 95% CI 1.63-2.33, p<0.001). and (OR=2.34, 95% Cl 1.78-3.09, p < 0.001) respectively.

**Cultural belief about HIV:** results from the logistic regression test indicated that attitude to HIV/AIDS was not significantly associated with HIV testing; X^2^ (1, N = 17467) = 2.182, p > 0.005 indicating the model was not able to distinguish between participant HIV testing. The result also showed that cultural belief about HIV does not explain the variance in HIV testing by women aged 15-49 years. Both Cox & Snell R2 and Nagelkerke R2 were 0%. But the model correctly classified 58.6% of the cases by HIV testing status. Cultural belief was not a predictor of HIV testing (OR=1.05, 95% CI 0.98-1.14, p>0.001).

**Socio-ecological factors:** multiple logistic regression analysis was performed using SPSS V27 to assess whether socio-ecological factors such as Knowledge about HIV/AIDS, attitude towards HIV/AIDS, perceived risk of HIV/AIDS, and cultural beliefs are predictors of HIV testing among women aged 15-49 years in Nigeria. The result showed that socio-ecological factors were predictors of HIV testing, indicating the model could distinguish between participant HIV testing. The model as a whole explained between 13.4% (Cox & Snell R2) to 18% (Nagelkerke R2) of the variance in HIV testing and correctly classified 68.1% of the cases. Table 3 shows the inferential statistics for each predictor variables in the model.

**Table 3:** logistic regression analysis predicting HIV test by socio-ecological factors

							95% confidence interval	
Predictor variable	β	S.E	Wald	df	Sig	Odds Ratio	Lower	Upper
Knowledge about HIV	1.339	0.035	1488.812	1	<0.001	3.813	3.559	4.086
Attitude to HIV	0.915	0.039	562.747	1	<0.001	2.496	2.314	2.692
perceived risk of HIV			55.302	2	<0.001			
perceived risk of HIV (1)	0.561	0.098	35.052	1	<0.001	1.787	1.475	2.166
perceived risk of HIV (2)	0.708	0.153	21.389	1	<0.001	2.029	1.503	2.739
Cultural belief	-0.148	0.041	13.046	1	<0.001	0.862	0.796	0.934
Constant	-3.047	0.094	1047.589	1	<0.001	0.048		

HIV: Human Immunodeficiency Virus

## Discussion

This study showed that knowledge of HIV/AIDS, attitude about HIV and perceived risk of HIV were significant predictors of HIV testing among the studied socio-ecological factors. The odds ratio for knowledge of HIV/AIDS is 3.81, which imply that those with good HIV knowledge are 3.81 times more likely to test for HIV than those with insufficient HIV knowledge. Those with a good attitude towards HIV are 2.50 times more likely to test for HIV. Respondents with medium and high perceived risk of HIV are 1.79 and 2.03 times more likely to test for HIV than those with low risk. On the contrary, respondents with good cultural belief are 0.862 times less likely to test for HIV while controlling for other factors in the model. Despite the low testing rate in this study, it was found that most of the women had good cultural beliefs but low knowledge of HIV, bad attitude to HIV and low risk of HIV. HIV knowledge was found in this study to be significantly associated with HIV testing, similar to other studies in different groups such as couples and women in relationships [13], and youths in Nigeria [15]. High knowledge of HIV is more likely to understand the importance of HIV and also perform HIV test as a preventive measure. High perceived risk of HIV was associated with HIV testing in this study, similar to the study among people who inject drugs in Iran [11]. People with high perceived risk will more likely test for HIV when they have adequate knowledge of the risk factors for HIV infection. A bad attitude in terms of stigma was associated with HIV testing in pregnant women attending ANC [12] and women of reproductive age in Abuja, Nigeria [17]. Women with good knowledge of HIV/AIDS, a good attitude towards HIV, and a high perceived risk were more likely to test for HIV, while those with a good cultural belief were less likely to test for HIV. This study found that socio-ecological factors were significantly associated with HIV testing in women aged 15-49 years in Nigeria.

There is a limitation in generalizing the result of this study because the dataset for the 2013 NDHS was used for the study because there was no response for HIV testing in the 2018 NDHS, a more recent survey. There is a decline in the prevalence of HIV from 3.4% in 2013 [1] to 1.4% in 2018 [3] and there is a lot of measures in place towards the prevention and control of HIV. Therefore, the result of this study may not be generalizable now.

## Conclusion

This study showed that good HIV knowledge, positive attitude to HIV and high perceived risk were predictors of HIV testing in women of childbearing age in Nigeria. The findings imply that improving the level of knowledge of HIV, improving attitude to HIV and risk assessment can improve HV testing in women aged 15-49 years in Nigeria. Increasing the HIV testing rate in these women will lead to a higher percentage of women living with HIV knowing their status and taking action accordingly to prevent transmission to their partners and children. HIV testing is the entry point for HIV treatment which can be used as prevention because treatment leads to a reduction in viral load and women with undetectable viral load will not transmit HIV to their partners and children as such the 95-95-95 target by 2025 [18,19] and the sustainable development goal of elimination of HIV as public health problem [20].

### What is known about this topic

- Educational status, perceived risk and HIV knowledge were predictors of HIV testing in women who were in a relationship with men in Nigeria;
- Educational status, higher wealth index, comprehensive HIV knowledge, HIV risk perception are associated with HIV testing among youths (males and females) in Nigeria;
- Educational status is significantly associated with HIV testing by pregnant women that attend the antenatal clinic in Nigeria.

### What this study adds

- Good comprehensive knowledge of HIV is positively predictive of HIV testing in women of childbearing age in Nigeria;
- Good attitude to HIV is positively predictive of HIV testing in women of childbearing age in Nigeria;
- High perceived risk of HIV is positively predictive of HIV testing in women of childbearing age in Nigeria.
